# Ferric Ion Diffusion for MOF-Polymer Composite with Internal Boundary Sinks

**DOI:** 10.3390/nano12050887

**Published:** 2022-03-07

**Authors:** Kirsten I. Louw, Bronwyn H. Bradshaw-Hajek, James M. Hill

**Affiliations:** UniSA STEM, University of South Australia, Mawson Lakes, SA 5095, Australia; kirsten.louw@mymail.unisa.edu.au (K.I.L.); jim.hill@unisa.edu.au (J.M.H.)

**Keywords:** diffusion, ferric ion sensor, MOF, finite difference, composite materials

## Abstract

Simple and economical ferric ion detection is necessary in many industries. An europium-based metal organic framework has selective sensing properties for solutions containing ferric ions and shows promise as a key component in a new sensor. We study an idealised sensor that consists of metal organic framework (MOF) crystals placed on a polymer surface. A two-dimensional diffusion model is used to predict the movement of ferric ions through the solution and polymer, and the ferric ion association to a MOF crystal at the boundary between the different media. A simplified one-dimensional model identifies the choice of appropriate values for the dimensionless parameters required to optimise the time for a MOF crystal to reach steady state. The model predicts that a large non-dimensional diffusion coefficient and an effective association with a small effective flux will reduce the time to steady-state. The effective dissociation is the most significant parameter to aid the estimation of the ferric ion concentration. This paper provides some theoretical insight for material scientists to optimise the design of a new ferric ion sensor.

## 1. Introduction

There are many situations in which it is important to monitor the concentration of ferric ions (trivalent iron cation or iron(III)) in a solution. For example, in environmental contexts, a high concentration of ferric ions promotes bacterial and algal growth, which can lead to the death of aquatic animals and plants [1,2]. In the mining industry, the concentration of ferric ions can affect copper and gold yield during mineral leaching, with both high and low concentrations producing adverse effects [3,4]. Other areas in which a knowledge of the ferric ion concentration is important include health and drinking water quality assurance [5,6].

This research is part of a larger project on lean mineral processing wherein a new sensor is being developed to detect ferric ions in a time-, cost-, and energy-efficient manner. Current ferric ion sensing methods are complex, use expensive equipment, suffer from interference from other ions, and are not adaptable to real time monitoring [7]. In addition, ferric ion concentrations are often determined indirectly [7]. As a consequence, a new ferric ion sensor that is stable, cost-effective, energy-efficient, and easy to use is desirable and would impact many sectors. In this paper, we present an idealised model of a ferric ion sensor. We propose a diffusion model of ferric ions through two different media with a sink located at the boundary separating the media.

Xu et al. [8] recently established the effectiveness of a europium-based metal organic framework (EuMOF) for ferric ion sensing. The crystalline EuMOF was suspended in an aqueous solution and tested against various concentrations of metal ions for changes in luminescence. Most MOF ferric ion sensors are intensity-based “turn-off” sensors where the intensity of light emitted by the sensor diminishes in the presence of ferric ions. However, the EuMOF [8] is bimodal, so changes in the emission ratio of two frequency peaks are linearly proportional to the ferric ion concentration, while the intensity of the emission is not as important. Metal ions such as Fe${}^{2+}$, Ag${}^{2+}$, Ca${}^{2+}$, and Zn${}^{2+}$ have little effect on the emission ratio, and consequently, the ferric ion selectivity of the EuMOF has promising sensing capabilities.

In [9] we investigated the van der Waals interaction energies and forces between a hydrated ferric ion and an EuMOF crystal pore. The Coulombic forces were not considered since ferric ions exist in highly acidic solutions, and the electrostatic forces are negligible [9]. The findings suggest that the hydrated ferric ion is attracted to the pore but does not enter due to steric interactions. This is advantageous from a practical point of view because the hydrated ferric ion can be “washed“ away and the sensor can be reused.

In this paper, we investigate how ferric ions diffuse through a solution and a polymer layer and interact with a MOF crystal located on the boundary between the solution and the polymer. One-dimensional diffusion through multiple layers has been studied before. Hickson et al. used a numerical approach to solving diffusion through multiple layers while considering various matching conditions at the boundary [10]. Carr and Turner [11] developed a semi-analytical method to address the complexities that arise for a large number of layers. An analytical solution exists for the one-dimensional problem with two layers (or *m* layers) for slabs, cylinders, and spheres [12].

A proposed new ferric ion sensor consists of a thin film of polymer coating a cut-away section of optic fibre and embedded with EuMOF crystals. In this scenario, the sensor will have MOF crystals distributed throughout the polymer composite and over its surface. In our model, we place an EuMOF crystal at the boundary between a solution and a polymer, and we investigate the changing concentration of ferric ions bound to the crystal. The paper provides important insight into ferric ion behaviour for a proposed sensor that is still under development and for which there is no readily available experimental data. Squires et al. [13] explore how the physical attributes of a sensor affect the flux onto the sensor, the rate at which substance of interest binds to the sensor, and the approximate times scales for the system to reach equilibrium. They present simple rules to equip the reader with a basic understanding of the environment and enable the design of a sensor better suited to the environment. Yariv [14] developed an advection–diffusion–reaction model of analytes binding to a sensor located on a solid surface adjacent to a shear flow of solution, using an equation first proposed in [13] to describe the concentration on the sensor. Here, we use a similar model to represent the association and disassociation of ferric ions to the EuMOF crystals.

In Section 2, we describe the geometry and the physical environment of the ferric ion sensor. We present a mathematical model that describes both the diffusion of ferric ions through an analyte solution and a polymer region, as well as the association of ferric ions to a MOF crystal located at the boundary between the two regions. Section 3 discusses outcomes of the model and the role and importance of each of the parameters.

## 2. Mathematical Modelling and Assumptions

**Figure 1:**
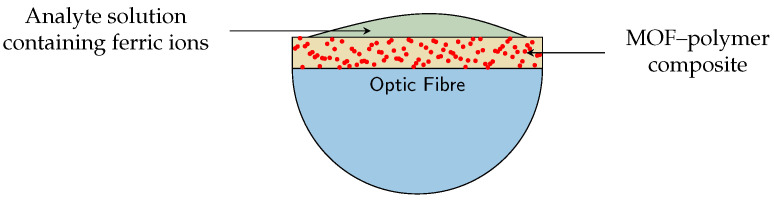
Two-dimensional cross-section of optic fibre with MOF–polymer thin film. Red dots represent MOF crystals in yellow polymer composite. Green represents solution being analysed.

One possible design for a ferric ion sensor is to suspend EuMOF crystals in a polymer composite to create a thin film on a cut away section of an optic fibre, as described by [15]. The ferric ions bind to the MOF pores on the crystal surface. A light pulse is sent through the MOF–polymer-coated optic fibre and a detector measures changes in luminosity. Changes in luminosity are indicative of the concentration of ferric ions in the solution. Figure 1 depicts a cross section of the optic fibre and MOF–polymer coating.

**Figure 2:**
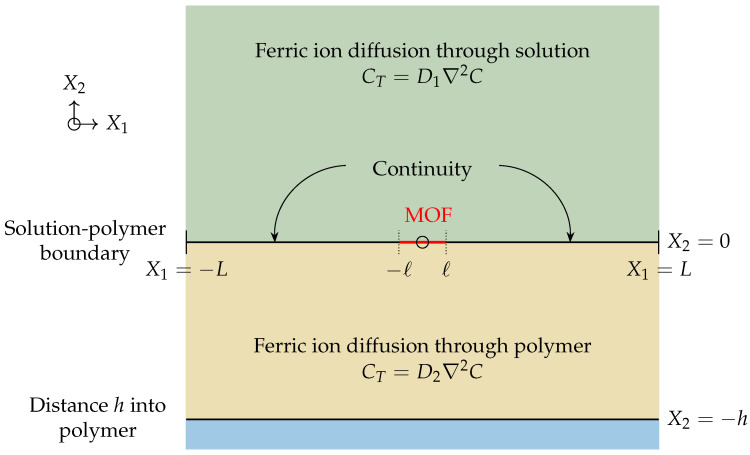
Two-dimensional system: $X_{1}$-axis is solution–polymer boundary, $X_{2}>0$ is distance into solution, and $X_{2}<0$ is into polymer composite.

Here, we are particularly interested in the diffusion of the ferric ions through the mixed media and their association with the MOF crystals in order to gain some insight into which physical parameters might be most important. As such, we study an idealised version of the device depicted in Figure 2. We model only the process of diffusion in the analyte solution, the association of ferric ions to a MOF crystal located at the interface between the polymer composite and the solution, and diffusion in the polymer composite. We position one MOF crystal at the interface between the solution and the polymer matrix. We do not consider the association of ferric ions with MOF crystals inside the polymer composite.

The MOF–polymer composite is exposed to a solution containing ferric ions, where the movement of ferric ions is governed by Brownian motion. We assume that the diffusivity of the ferric ions is constant through the solution, denoted by $D_{1}$. Since, in practice, the polymer is kept hydrated and the ferric ions do not enter the MOF pores, we assume that the diffusivity in the polymer is also constant, $D_{2}<D_{1}$, and the Vrentas–Duda theory is not applicable. As a consequence, the movement of ferric ions in both the solution and the polymer is assumed to be governed by the conventional diffusion equation with distinct diffusivities,

$$\begin{array}{cc} \frac{\partial }{\partial T}C\left(T,X\right) & =D_{1}\nabla^{2}C\left(T,X\right),\;X_{2}\geq 0,\\ \frac{\partial }{\partial T}C\left(T,X\right) & =D_{2}\nabla^{2}C\left(T,X\right),\;X_{2}<0, \end{array}$$

where C(T,X) is the concentration of ferric ions, and the origin is located at the centre of the MOF strip so that the $X_{1}$-axis coincides with the solution–polymer boundary. Here, $\nabla^{2}$ is the usual Laplacian in two dimensions. The ferric ion concentration profile in the analyte solution is given by C(T,X) for $X_{2}\geq 0$, and the concentration in the polymer is given by C(T,X) for $X_{2}<0$. The time since the sensor’s exposure to the solution is given by *T*.

To ensure that continuity is maintained for boundaries shared by two media, we include two additional conditions. The ferric ion concentration at the boundary is assumed to be the same when approaching from above or below the boundary, and the flux out of the solution is equivalent to the flux into the polymer [10],

$$C_{+}=C_{-},\;D_{1}\frac{\partial C_{+}}{\partial X_{2}}=D_{2}\frac{\partial C_{-}}{\partial X_{2}},\;X_{2}=0.$$

Far from the sensor in the direction of the analyte solution, we assume a far field boundary condition, where the ferric ion concentration remains constant and equal to that of the bulk solution $C_{0}$. For numerical purposes, we set the location of this condition to be at $X_{2}=L$, so that

$$C\left(T,X_{1},L\right)=C_{0},\;X_{2}=L,$$

where *L* is assumed to be a large distance away from the solution–polymer boundary. We also assume no flux at a horizontal distance *L* away from the MOF centre (symmetry) and at the bottom boundary of the polymer composite,

$$\frac{\partial C}{\partial X_{1}}=0,\;X_{1}=\pm L,\;\frac{\partial C}{\partial X_{2}}=0,\;X_{2}=-h.$$

The flux condition at the solution–MOF interface is given by

$$D_{1}\frac{\partial C_{+}}{\partial X_{2}}=a\left[k_{\mathrm{on}}C\;\left(B_{0}-B\right)-k_{\mathrm{off}}B\right],\;\left|X_{1}\right|\leq \ell ,\;X_{2}=0,$$

where $B(T,X_{1})$ is the occupancy of the MOF pores by the ferric ions, $k_{\mathrm{on}}$ is the association rate compared to the ferric ion concentration, $k_{\mathrm{off}}$ is the dissociation rate, $B_{0}$ is the number of MOF pores available for ferric ion occupation, and *a* is a constant. Due to steric effects, the ferric ion only associates at the MOF pore’s entrance and does not diffuse into the MOF pore. The first term captures the association of ferric ions to the MOF crystal, and this term vanishes when the MOF crystal is completely occupied. The second term captures the dissociation of ferric ions from the MOF pore.

We assume that ferric ions only associate and dissociate from the MOF pores in the $X_{2}$-direction and impose a no-flux boundary condition to the sides of the MOF crystal,

$$\frac{\partial C}{\partial X_{1}}=0,\;\left|X_{1}\right|=\ell ,\;X_{2}=0.$$

The occupancy of the MOF pores is governed by the pseudo-ordinary differential equation (ODE),

$$\frac{\partial B}{\partial T}=k_{\mathrm{on}}C\;\left(B_{0}-B\right)-k_{\mathrm{off}}B,\;\left|X_{1}\right|\leq \ell ,\;X_{2}=0.$$

In addition, since $B_{0}$ is the number of MOF pores available, $B\left(T,X_{1}\right)\in \left[0,B_{0}\right]$ and the total ferric ion occupancy in the MOF crystal is,

$$B_{tot}\left(T\right)=\int_{-\ell }^{\ell }B\left(T,X_{1}\right)\;dX_{1}\leq 2\;\ell \;B_{0}.$$

The assumed initial conditions are

$$\begin{array}{cc} C(0,X) & =C_{0},\;X_{2}\geq 0,\;C\left(0,X\right)=0,\;X_{2}<0,\\ B(0,X_{1}) & =0,\;|X_{1}|\leq \ell ,\;X_{2}=0. \end{array}$$

### 2.1. Dimensionless Two-Dimensional Model

We non-dimensionalise distance with the half length of the MOF strip, *ℓ*, the ferric ion concentration with the bulk concentration, $C_{0}$, and the MOF binding site occupancy with the maximum occupancy, $B_{0}$. There are two options for the time scale: a diffusive time-scale, where $\tau =\ell^{2}/D_{1}$, or an association time-scale, where $\tau =1/C_{0}k_{\mathrm{on}}$. At this stage, we non-dimensionalise time with τ without specifying which time-scale so that the system of equations can be written

(1) $$\begin{array}{cc} \frac{\partial c}{\partial t} & =s_{1}\nabla^{2}c,\;x_{2}\geq 0,\;\frac{\partial c}{\partial t}=s_{1}D\nabla^{2}c,\;x_{2}<0, \end{array}$$

(2) $$\begin{array}{cc} \frac{\partial b}{\partial t} & =s_{2}\left[c\;\left(1-b\right)-s_{3}\;b\right],\;\left|x_{1}\right|\leq 1,\;x_{2}=0, \end{array}$$

where $D=D_{2}/D_{1}$, $s_{1}=D_{1}\tau /\ell^{2}$, $s_{2}=C_{0}k_{\mathrm{on}}\tau$ and $s_{3}=k_{\mathrm{off}}/C_{0}k_{\mathrm{on}}$ are non-dimensional parameters. If the problem is scaled with the diffusive time-scale, then $s_{1}=1$. If the problem is scaled with the association time-scale, then $s_{2}=1$.

The continuity conditions at the solution–polymer boundary become

(3) $$c_{+}=c_{-},\;\frac{\partial c_{+}}{\partial x_{2}}=D\frac{\partial c_{-}}{\partial x_{2}},\;x_{2}=0.$$

Far away from the solution–polymer boundary, the boundary condition becomes

(4) $$c\left(t,x_{1},L/\ell \right)=1,\;x_{2}=L/\ell ,$$

while the no-flux boundary conditions at the sides and at the polymer–optic fibre boundary become

(5) $$\frac{\partial c}{\partial x_{1}}=0,\;x_{1}=\pm L/\ell ,\;\frac{\partial c}{\partial x_{2}}=0,\;x_{2}=-h/\ell .$$

At the solution–MOF boundary,

(6) $$s_{1}\frac{\partial c_{+}}{\partial x_{2}}=s_{4}\;s_{2}\left[c\;\left(1-b\right)-s_{3}\;b\right],\;\left|x_{1}\right|\leq 1,\;\;x_{2}=0,$$

where $s_{4}=a\;B_{0}/\ell \;C_{0}$ is a non-dimensional constant, and the no-flux boundary conditions at the sides of the MOF crystal become

(7) $$\frac{\partial c}{\partial x_{1}}=0,\;\left|x_{1}\right|=1.$$

The initial conditions and the total MOF pore occupancy are

(8) $$\begin{array}{cc} c(0,x) & =1,\;x_{2}\geq 0,\;c\left(0,x\right)=0,\;x_{2}<0, \end{array}$$

(9) $$\begin{array}{cc} b(0,x_{1}) & =0,\;|x_{1}|\leq 1,\;x_{2}=0 \end{array}$$

(10) $$\begin{array}{cc} b_{tot}\left(t\right) & =\int_{-1}^{1}b\left(t,x_{1}\right)\;dx_{1}\leq 2. \end{array}$$

### 2.2. One-Dimensional Model

**Figure 3:**
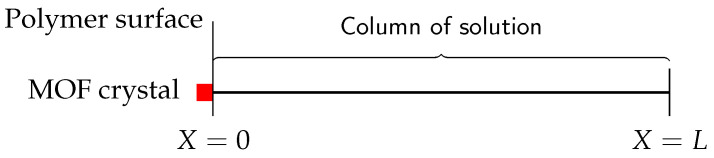
One-dimensional representation of ferric ions diffusing through a column of solution with MOF crystal at the left boundary.

To obtain a preliminary indicator of the behaviour of the system and the importance of the various parameters, we further simplify this model. Figure 3 shows a simpler one-dimensional system, where the MOF crystal is represented by a red square, and diffusion in the polymer composite region is completely ignored.

The concentration of ferric ions is assumed to be only a function of two variables, C(T,X), and the MOF pore occupancy is assumed to depend only on time, B(T),

$$\begin{array}{cc} \frac{\partial C}{\partial T} & =D_{1}\frac{\partial^{2}C}{\partial X^{2}},\;X\geq 0,\\ \frac{\partial B}{\partial T} & =k_{\mathrm{on}}\;C\left(B_{0}-B\right)-k_{\mathrm{off}}B,\;X=0. \end{array}$$

The corresponding flux condition at the boundary X=0 is given by

$$D_{1}\frac{\partial C}{\partial X}=a\left[k_{\mathrm{on}}\;C\left(B_{0}-B\right)-k_{\mathrm{off}}B\right],$$

and the far field condition is $C\left(T,L\right)=C_{0}$, with initial conditions $C\left(0,X\right)=C_{0}$ and B(0)=0.

### 2.3. Dimensionless One-Dimensional Model

We non-dimensionalise concentration, occupancy, length, and time in the same way as before, where *ℓ* is a characteristic length representative of the MOF crystal size so that the system of equations becomes

(11) $$\frac{\partial c}{\partial t}=s_{1}\;\frac{\partial^{2}c}{\partial x^{2}},\;x\geq 0,\;\frac{\partial b}{\partial t}=s_{2}\left[c\;\left(1-b\right)-s_{3}\;b\right],\;x=0,$$

where the $s_{i}$ are as previously defined. The non-dimensional flux condition at the boundary x=0 is

(12) $$s_{1}\frac{\partial c}{\partial x}=s_{4}\;s_{2}\left[c\;\left(1-b\right)-s_{3}\;b\right],$$

while the non-dimensional far field condition at x=L/ℓ becomes

(13) c(t,L/ℓ)=1,

and the initial conditions are given by

(14) c(0,x)=1,b(0)=0.

## 3. Results and Discussions

In this section, we discuss the results for the diffusion of ferric ions through the two regions and the ferric ion occupancy in the MOF pores. We vary the dimensionless constants, $s_{i}$ and *D*, to analyse how the parameters affect ferric ion diffusion and MOF occupancy.

The models are solved numerically, as no analytical solution exists for this system, using purpose written code in Matlab. First, we examine the dimensionless one-dimensional model where the equation for b(t) is solved using a Runga–Kutta method, and the diffusion equation for c(t,x) is solved with an implicit Crank–Nicolson scheme. Second, we examine the dimensionless two-dimensional model where the equation for $b(t,x_{1})$ is solved using a Runga–Kutta method, and the diffusion equation for c(t,x) is solved with a forward-time-centred-space finite difference scheme.

Further details about the numerical schemes for the dimensionless one-dimensional and two-dimensional models are presented in Appendix A and Appendix B. In addition, the authors have tested some extremely simple cases; for example, if the MOF crystal is not present. However, these results are trivial and are not included here.

### 3.1. Dimensionless One-Dimensional Model

**Figure 4:**
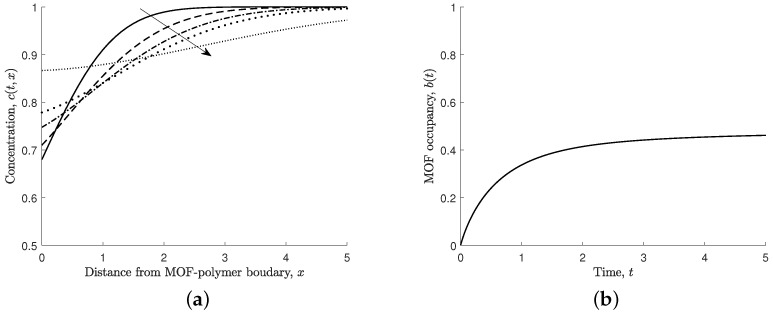
(**a**) Non-dimensional concentration of ferric ions in solution, c(t,0). Arrow indicates increasing time, t=0.5,1,1.5,2,5. (**b**) Non-dimensional MOF occupancy, b(t). Here, $s_{1}=s_{2}=s_{3}=s_{4}=1$.

Figure 4a depicts the time evolution of the ferric ion concentration in solution, and Figure 4b shows the MOF occupancy. Ferric ions in the solution initially associate rapidly to the MOF crystal, reducing the concentration in the solution near the MOF crystal. Over time, the ferric ion concentration returns to the steady-state far-field condition, as shown in Figure 4a. We note that the MOF crystal’s occupancy initially increases rapidly and then slowly increases until steady state is reached, as shown in Figure 4b.

In the following subsections, we describe the impact of varying parameters. In all cases for the one-dimensional scheme, Δx=0.1 and Δt=0.005, and we set L/ℓ=10.

#### 3.1.1. Varying the Non-Dimensional Diffusion Coefficient, $s_{1}$

**Figure 5:**
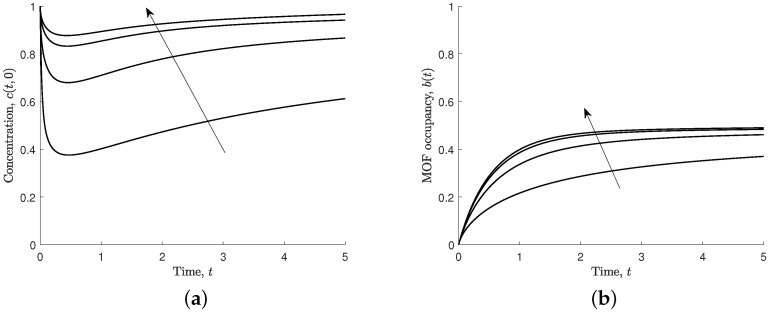
Results for different values of non-dimensional diffusion, $s_{1}=0.1,1,5,10$, and $s_{2}=s_{3}=s_{4}=1$. Arrows indicate the direction of increasing $s_{1}$. (**a**) Non-dimensional concentration of ferric ions at MOF–polymer boundary, c(t,0). (**b**) Non-dimensional MOF occupancy, b(t).

Figure 5 shows the effect of varying the non-dimensional diffusion coefficient ($s_{1}=D_{1}\tau /\ell^{2}$) on the ferric ion concentration in the solution at the MOF crystal and the MOF occupancy. A smaller non-dimensional diffusion coefficient causes the concentration of ferric ions in solution to be more significantly reduced near the MOF boundary, and, following this initial reduction, it takes longer for the concentration in the solution to recover towards the steady-state concentration. A larger non-dimensional diffusion coefficient results in less reduction near the boundary because ferric ions can more readily diffuse from the column of solution. MOF occupancy reaches close to steady-state sooner with a high non-dimensional diffusion coefficient.

#### 3.1.2. Varying the Effective Association Parameter, $s_{2}$

**Figure 6:**
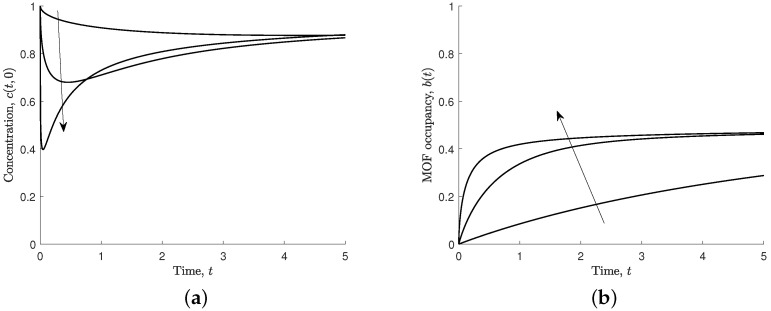
Results for different values of effective association, $s_{2}=0.1,1,10$, and $s_{1}=s_{3}=s_{4}=1$. Arrows indicate the direction of increasing $s_{2}$. (**a**) Non-dimensional concentration of ferric ions at MOF–polymer boundary, c(t,0). (**b**) Non-dimensional MOF occupancy, b(t).

Increasing the effective association parameter ($s_{2}=C_{0}k_{\mathrm{on}}\tau$) means that ferric ions associate to the MOF crystal faster than they diffuse through the solution. This results in the MOF occupancy reaching the steady state sooner, as shown in Figure 6b. When $s_{2}$ is large, the concentration near the solution–polymer boundary is reduced very quickly, followed by a slow increase as ferric ions diffuse from the rest of the region, as shown in Figure 6a.

Upon exposure of the MOF to the solution, the concentration at the boundary varies significantly for different values of $s_{2}$. However, at large times, the ferric ion concentration and the MOF occupancy is largely independent of $s_{2}$.

#### 3.1.3. Varying the Effective Dissociation Parameter, $s_{3}$

**Figure 7:**
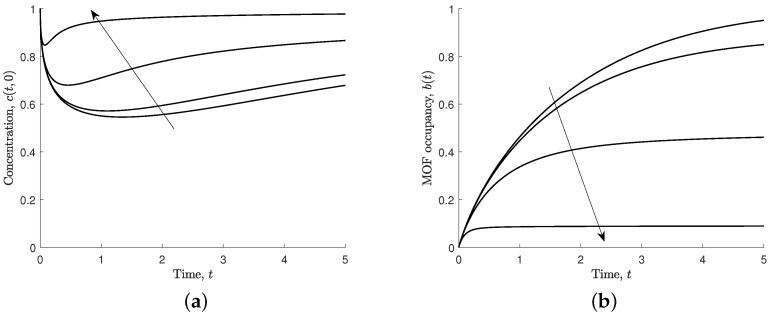
Results for different values of effective dissociation, $s_{3}=0,0.1,1,10$, and $s_{1}=s_{2}=s_{4}=1$. Arrows indicate the direction of increasing $s_{3}$. (**a**) Non-dimensional concentration of ferric ions at MOF–polymer boundary, c(t,0). (**b**) Non-dimensional MOF occupancy, b(t).

The effective dissociation parameter ($s_{3}=k_{\mathrm{off}}/C_{0}k_{\mathrm{on}}$) captures the ferric ions that dissociate from the MOF crystal. We have investigated various values of $s_{3}$, including $s_{3}=0$, which represents the situation when ferric ions can only associate—see Figure 7.

Increasing the effective dissociation parameter means that the ferric ions dissociate more readily and the non-dimensional steady-state MOF pore occupancy can be calculated from Equation (11),

(15) $$b_{\mathrm{eq}}=b\left(t\to \infty \right)=\frac{1}{1+s_{3}}.$$

A similar expression for the number of effective bound receptors at equilibrium is given in Squires et al. [13], with a different combination of parameters. (The present authors believe that the equilibrium constant $K_{D}$ in Squires et al. [13] should be defined as $K_{D}=k_{\mathrm{off}}/k_{\mathrm{on}}$ rather than $K_{D}=k_{\mathrm{on}}/k_{\mathrm{off}}$ as stated. This also aligns with working in their Box 1).

In the steady state, the occupancy of the pores is lower for larger values of the effective dissociation parameter, and the steady state is reached more quickly. In the case when the ferric ions cannot dissociate, that is $s_{3}=0$, the steady state MOF occupancy is equal to its maximum value (b(t→∞)=1), and the concentration of ferric ions at the MOF–solution boundary approaches the bulk concentration at large times.

#### 3.1.4. Varying the Effective Flux Parameter, $s_{4}$

**Figure 8:**
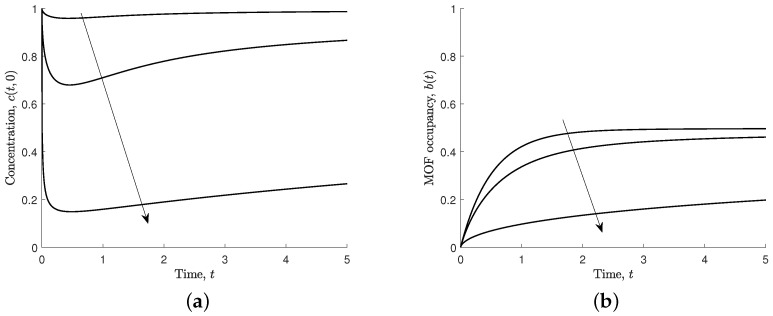
Results for different values of effective flux, $s_{4}=0.1,1,10$, and $s_{1}=s_{2}=s_{3}=1$. Arrows indicate the direction of increasing $s_{4}$. (**a**) Non-dimensional concentration of ferric ions at MOF–polymer boundary, c(t,0). (**b**) Non-dimensional MOF occupancy, b(t).

An important consequence of the flux condition is the time taken for the MOF crystal to approach steady state. Increasing the effective flux parameter ($s_{4}=aB_{0}/\ell C_{0}$) increases the time taken to reach the steady state (see Figure 8b). The increase affects ferric ion concentration at the MOF crystal boundary, as ferric ions quickly associate to the MOF crystal and the ferric ions do not diffuse fast enough for the MOF crystal to reach steady state in a timely manner, as shown in Figure 8a.

The delay to reaching steady state is attributed to ferric ions associating to the MOF crystal faster and quickly depleting the ferric ions in the solution. A comparable mechanism is observed when increasing the effective association parameter, $s_{2}$. The effective association parameter is a MOF characteristic, and an increase results in a shorter time for the MOF crystal to reach steady state (see Figure 6b). The ferric ion concentration at the MOF boundary is largely unchanged after t=4, as shown in Figure 6a. However, the effective flux parameter is a ferric ion characteristic, and an increase in this parameter results in a longer time for the MOF crystal to reach steady state, as shown in Figure 8b. The ferric ion concentration at the MOF boundary is very sensitive to the increase in the effective flux parameter, as seen by the long depletion time for a larger parameter value in Figure 8a.

### 3.2. Dimensionless Two-Dimensional Model

In this section, we return to the dimensionless two-dimensional model and to the primary aim of the paper, which is to investigate ferric ion diffusion through a solution into a polymer composite with a MOF sink located at the boundary between the two media.

**Figure 9:**
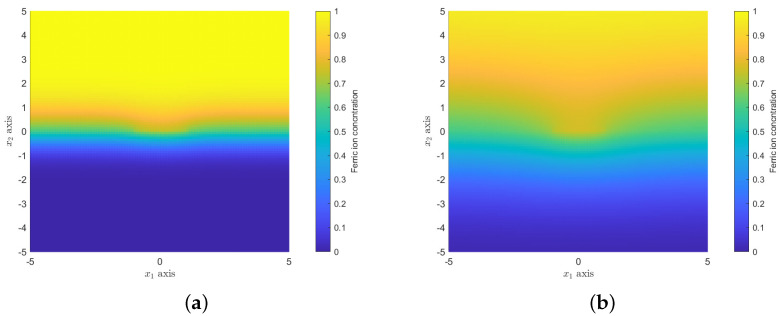
Non-dimensional concentration of ferric ions across two media $c(t,x_{1},x_{2})$: (**a**) at time t=0.5; (**b**) at time t=5.

**Figure 10:**
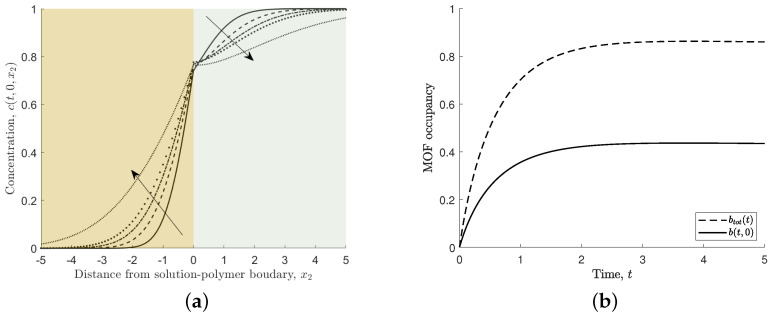
(**a**) Non-dimensional concentration of ferric ions through MOF-mid point across two media, $c(t,0,x_{2})$. Arrow indicates the direction of increasing time, t=0.5,1,1.5,2,5. (**b**) Non-dimensional MOF occupancy, b(t,0) and $b_{tot}\left(t\right)$. Here, $s_{1}=s_{2}=s_{3}=s_{4}=1$, and D=0.5.

Figure 9 shows the ferric ion concentration for the domain $-5\leq x_{1}\leq 5,\;-5\leq x_{2}\leq 5$, where $x_{2}<0$ is the concentration in the polymer and $x_{2}\geq 0$ is the concentration in the solution. The MOF crystal is located at $|x_{1}|\leq 1$ and $x_{2}=0$. Figure 9a,b show the ferric ion profiles at time t=0.5 and 5, respectively. In all cases for the numerical two-dimensional scheme, $\Delta x_{1}=\Delta x_{2}=0.1$ and Δt=0.0005, and D=0.5 (unless otherwise stated) and L/ℓ=h/ℓ=10. Figure 10a shows the ferric ion concentration along the line $x_{1}=0$, which shows the changes through the mixed media over time. In comparison to the one-dimensional model (Figure 4a), the two-dimensional model requires additional time to reach steady state. This is due to the size of the MOF crystal and the time taken to reach steady state in the two-media system. The MOF occupancy at the centre of the MOF (b(t,0)) and the total MOF occupancy over the whole MOF crystal (Equation (10)) is shown in Figure 10b. By comparing Figure 10b with Figure 4b, we see that the MOF occupancy as predicted by the two-dimensional model is very similar to that predicted by the one-dimensional model. However, the two-dimensional model includes the effect of the polymer on ferric ion diffusion and greater MOF occupancy potential.

#### 3.2.1. Varying the Non-Dimensional Parameters

Variation of the dimensionless constants has the same effect on the two-dimensional MOF occupancy b(t,0) profile when compared to the one-dimensional MOF occupancy b(t). The two-dimensional ferric ion concentration at the centre of the MOF crystal behaves in a similar way to that of the one-dimensional ferric ion profile shown in Figure 4a. However, more time is needed for the ferric ion concentration to reach steady state due to a larger MOF crystal surface area.

**Figure 11:**
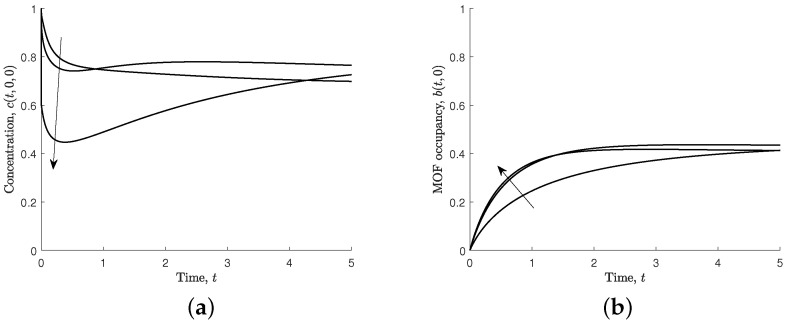
Results for different values of non-dimensional diffusion, $s_{1}=0.1,1,5$, $s_{2}=s_{3}=s_{4}=1$, and D=0.5. Arrows indicate the direction of increasing $s_{1}$. (**a**) Non-dimensional concentration of ferric ions through MOF mid-point across two media, c(t,0,0). (**b**) Non-dimensional MOF occupancy, b(t,0).

Figure 11 shows the effect of varying the non-dimensional diffusion coefficient ($s_{1}=0.1,1,5$). For all values of $s_{1}$, the concentration in the solution near the MOF surface initially decreases before increasing towards the steady state value. For larger values of $s_{1}$, the decrease is more significant, which is in contrast to the one-dimensional results, as shown in Figure 5.

The changes in the other non-dimensional parameters ($s_{2},s_{3},s_{4}$) produce similar results to those discussed for the one-dimensional model. Figures showing the details can be found in Appendix C.

#### 3.2.2. Varying the Relative Diffusion Coefficient, *D*

**Figure 12:**
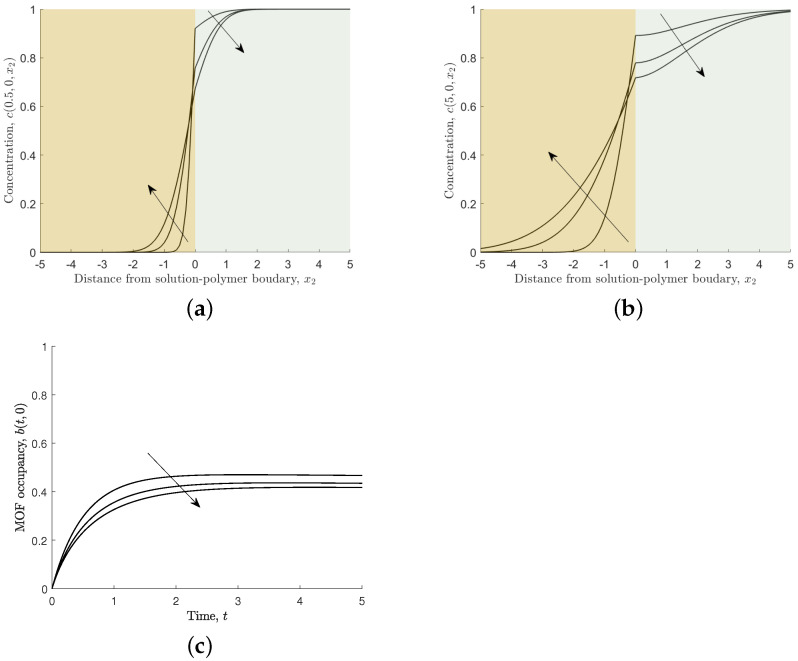
Results for different values of relative diffusion coefficient, D=0.1,0.5,1, and $s_{1}=s_{2}=s_{3}=s_{4}=1$. Arrows indicate the direction of increasing *D*. (**a**,**b**) Non-dimensional concentration of ferric ions through MOF mid-point across two media, $c(t,0,x_{2})$ at time t=0.5 and 5, respectively. (**c**) Non-dimensional MOF occupancy, b(t,0).

If ferric ion diffusivity in the solution and the polymer is the same, then the relative diffusion coefficient is unity, $D=D_{2}/D_{1}=1$. In practice, the diffusion coefficient of ferric ions in the solution will exceed that in the polymer and the larger the difference the smaller the relative diffusion coefficient. Figure 12 shows the effects of varying the relative diffusion coefficient.

Decreasing the relative diffusion coefficient means that ferric ions take longer to diffuse in the polymer and away from the solution–polymer boundary. This affords the ferric ions more opportunity to associate to the MOF crystal. Figure 12c shows that the occupancy in the crystal is highest in the case of the smallest relative diffusion coefficient.

## 4. Conclusions

In this paper, we have modelled a proposed new ferric ion sensor. We first examined a two-dimensional model to describe the diffusion of the ions through a solution and into a polymer layer, as well as their attachment to an EuMOF crystal located at the boundary of the two media. We then examined a simplified model that includes diffusion through the solution only, together with attachment at the MOF crystal. The solutions to the corresponding non-dimensional models are then analysed to investigate the importance of the various non-dimensional parameters.

The one-dimensional model indicates that at steady state, the occupancy of the MOF crystal is given by Equation (15), and this occupancy depends only on the initial concentration of ferric ions in the solution and the effective dissociation constant, $s_{3}=k_{\mathrm{off}}/C_{0}k_{\mathrm{on}}$. As a consequence, if the steady-state occupancy, $B_{\mathrm{eq}}=B\left(t\to \infty \right)$, can be measured with the new sensor, and the association rate $k_{\mathrm{on}}$, dissociation rate $k_{\mathrm{off}}$, and maximum occupancy $B_{0}$ are known, then the concentration of ferric ions in solution can be calculated (in dimensional terms) using

(16) $$C_{0}=\frac{k_{\mathrm{off}}B_{\mathrm{eq}}}{k_{\mathrm{on}}\left(B_{0}-B_{\mathrm{eq}}\right)}.$$

In practice, it is most likely to be useful to minimise the time taken to reach steady state so that sensing of multiple samples can be carried out quickly and efficiently. To achieve this, it would be helpful to have a large non-dimensional diffusion coefficient $s_{1}$ and effective association constant $s_{2}$ with a small effective flux constant $s_{4}$.

While the two-dimensional model is more representative of the proposed sensor and provides more information about the behaviour of the ferric ions in the solution and the polymer, many of the broad behaviours and impacts of changing the system parameters are captured by the one-dimensional model. The relative diffusion coefficient *D* suggests that the polymer composite should hinder ferric ion diffusion in that region. A final sensor design is likely to include MOF crystals embedded within the polymer matrix rather than on the surface only, so the polymer composite should be designed so that the relative diffusion coefficient is close to unity in order for ferric ions to associate to MOF crystals inside the polymer composite. This situation is not examined here but will be the subject of future work. In this case, a two-dimensional model is likely to provide more insight.

## Figures and Tables

**Figure A1:**
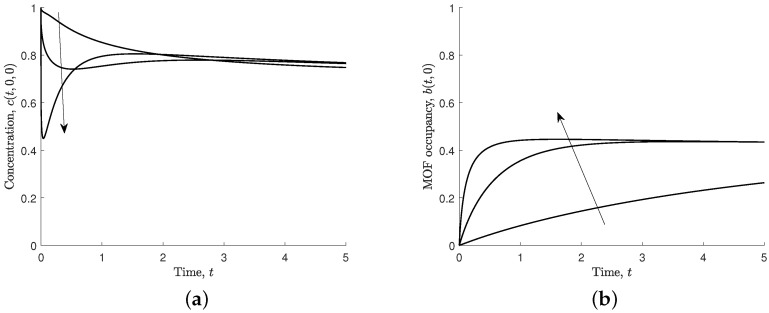
Results for different values of effective association, $s_{2}=0.1,1,10$, $s_{1}=s_{3}=s_{4}=1$, and D=0.5. Arrows indicate the direction of increasing $s_{2}$. (**a**) Non-dimensional concentration of ferric ions through MOF-mid point across two media, $c(t,0,x_{2})$. (**b**) Non-dimensional MOF occupancy, b(t,0).

**Figure A2:**
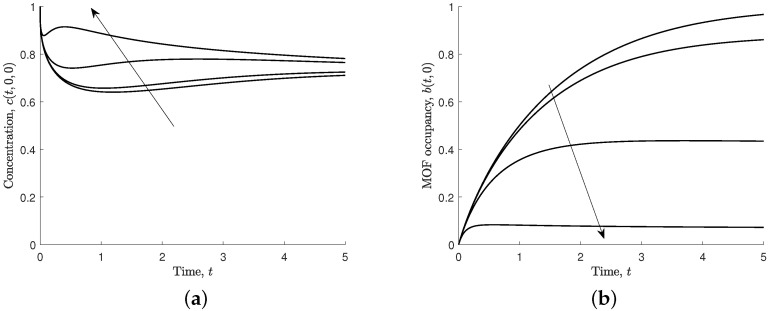
Results for different values of effective dissociation, $s_{3}=0,0.1,1,10$, $s_{1}=s_{2}=s_{4}=1$, and D=0.5. Arrows indicate the direction of increasing $s_{3}$. (**a**) Non-dimensional concentration of ferric ions through MOF-mid point across two media, $c(t,0,x_{2})$. (**b**) Non-dimensional MOF occupancy, b(t,0).

**Figure A3:**
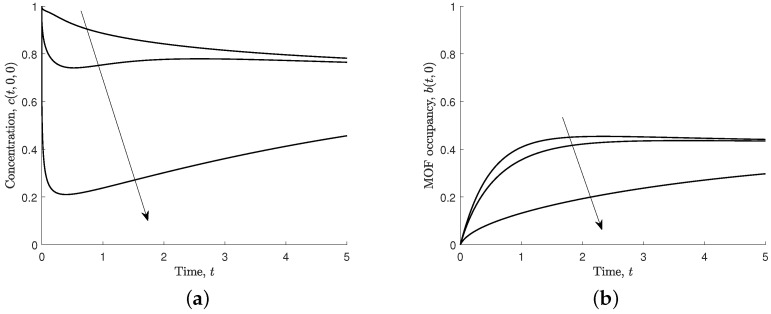
Results for different values of effective capacity, $s_{4}=0.1,1,10$, $s_{1}=s_{2}=s_{3}=1$, and D=0.5. Arrows indicate the direction of increasing $s_{4}$. (**a**) Non-dimensional concentration of ferric ions through MOF-mid point across two media, $c(t,0,x_{2})$. (**b**) Non-dimensional MOF occupancy, b(t,0).

## Data Availability

Numerical details for this study are presented in the Appendices. Data sharing is not applicable for this article.

## Abbreviations

The following abbreviations are used in this manuscript:

MOF	Metal organic framework
EuMOF	Europium based metal organic framework
ODE	Ordinary differential equation

## Appendix A. Algorithm for Dimensionless One-Dimensional Model

The functions describing the ferric ion concentration c(t,x) and MOF occupancy b(t) are discritised, and the following definitions are used,

$$c\left(t_{n},x_{i}\right)=c_{i}^{n},\;c\left(t,0\right)=c_{0}^{n},\;c\left(t,L/\ell \right)=c_{I}^{n},\;b\left(t_{n}\right)=b^{n}.$$

We first update the value of the MOF occupancy $b^{n+1}$ so that we can use the updated value to calculate the ferric ion concentration $c_{0}^{n+1}$ at x=0. A Runge–Kutta 4 scheme is used

$$\begin{array}{cc} f(y) & =s_{2}\;c_{0}^{n}\left(1-y\right)-s_{2}\;s_{3}y,\\ k_{1} & =\Delta t\;f\left(b^{n}\right),\;k_{2}=\Delta t\;f\left(b^{n}+\frac{k_{1}}{2}\right),\\ k_{3} & =\Delta t\;f\left(b^{n}+\frac{k_{2}}{2}\right),\;k_{4}=\Delta t\;f\left(b^{n}+k_{3}\right). \end{array}$$

At time step n+1, the MOF occupancy is

$$b^{n+1}=b^{n}+\frac{1}{6}\left(k_{1}+2k_{2}+2k_{3}+k_{4}\right),$$

where $b\left(0\right)=b^{0}=0$.

Next, we update the ferric ion concentration throughout the solution using the Crank–Nicolson central forwards time, centred space. The following difference formulas for the first and second derivatives were used

$$\begin{array}{cc} \; & c\left(t,x\right)=\frac{1}{2}\left(c_{i}^{n}+c_{i}^{n+1}\right),\\ \; & \frac{\partial }{\partial x}c\left(t,x\right)=\frac{1}{2}\left[\frac{c_{i+1}^{n}-c_{i-1}^{n}}{2\Delta x}+\frac{c_{i+1}^{n+1}-c_{i-1}^{n+1}}{2\Delta x}\right],\;\frac{\partial }{\partial t}c\left(t,x\right)=\frac{c_{i}^{n+1}-c_{i}^{n}}{\Delta t}\\ \; & \frac{\partial^{2}}{\partial x^{2}}c\left(t,x\right)=\frac{1}{2}\left[\frac{c_{i+1}^{n}-2c_{i}^{n}+c_{i-1}^{n}}{\Delta x^{2}}+\frac{c_{i+1}^{n+1}-2c_{i}^{n+1}+c_{i-1}^{n+1}}{\Delta x^{2}}\right]. \end{array}$$

Substituting these formulas into the ferric ion diffusion equation gives

$$\frac{c_{i}^{n+1}-c_{i}^{n}}{\Delta t}=\frac{s_{1}}{2}\left[\frac{c_{i+1}^{n}-2c_{i}^{n}+c_{i-1}^{n}}{\Delta x^{2}}+\frac{c_{i+1}^{n+1}-2c_{i}^{n+1}+c_{i-1}^{n+1}}{\Delta x^{2}}\right].$$

We collect terms with time step n+1 on the left and *n* on the right hand side of the equation so that the general scheme is

$$-\alpha \;c_{i+1}^{n+1}+\left(2+2\alpha \right)c_{i}^{n+1}-\alpha \;c_{i-1}^{n+1}=\alpha \;c_{i+1}^{n}+\left(2-2\alpha \right)c_{i}^{n}+\alpha \;c_{i-1}^{n},$$

where $\alpha =s_{1}\Delta t/\Delta x^{2}$. The boundary conditions concerning the ferric ion concentration need to be incorporated, and so, the flux at the MOF–polymer boundary (Equation (12)) becomes

$$s_{1}\left[c_{1}^{n}-c_{-1}^{n}+c_{1}^{n+1}-c_{-1}^{n+1}\right]=2s_{4}s_{2}\Delta x\left(c_{0}^{n}+c_{0}^{n+1}\right)\left(1-b^{n+1}\right)-4\Delta xs_{4}s_{2}s_{3}\;b^{n+1}.$$

Now, adjust the general scheme at and near the boundaries:

for i=0, the MOF–polymer boundary,

$$\begin{array}{cc} -2\alpha \;c_{1}^{n+1}+[2+2\alpha & +2\alpha s_{4}s_{2}\Delta x\left(1-b^{n+1}\right)/s_{1}]c_{0}^{n+1}\\ \; & =2\alpha \;c_{1}^{n}+\left[2-2\alpha -2\alpha s_{4}s_{2}\Delta x\left(1-b^{n+1}\right)/s_{1}\right]c_{0}^{n}+4\Delta xs_{2}s_{3}s_{4}\;b^{n+1}/s_{1}, \end{array}$$

for i=I, far-field boundary (Equation (13)),

$$c_{I}^{n}=1,\;\forall n,$$

for i=I−1,

$$\left(2+2\alpha \right)c_{I-1}^{n+1}-\alpha \;c_{I-2}^{n+1}=\left(2-2\alpha \right)c_{I-1}^{n}+\alpha \;c_{I-2}^{n}+2\alpha .$$

The finite difference scheme can be written as a linear system,

$$L{\underline{c}}^{n+1}=R{\underline{c}}^{n}+A,$$

where

$$L=\left[\begin{array}{ccccccccc} 2+2\alpha +2\alpha s_{4}s_{2}\Delta x\left(1-b^{n+1}\right)/s_{1} & -\alpha & 0 & 0 & \cdots & 0 & 0 & 0 & 0\\ -\alpha & 2+2\alpha & -\alpha & 0 & \cdots & 0 & 0 & 0 & 0\\ \ddots & \ddots & \ddots & \; & \cdots & 0 & 0 & 0 & 0\\ 0 & 0 & 0 & 0 & \cdots & -\alpha & 2+2\alpha & -\alpha & 0\\ 0 & 0 & 0 & 0 & \cdots & 0 & -\alpha & 2+2\alpha & 0\\ 0 & 0 & 0 & 0 & \cdots & 0 & 0 & 0 & 1 \end{array}\right],$$

$$R=\left[\begin{array}{ccccccccc} 2-2\alpha -2\alpha s_{4}s_{2}\Delta x\left(1-b^{n+1}\right)/s_{1} & \alpha & 0 & 0 & \cdots & 0 & 0 & 0 & 0\\ \alpha & 2-2\alpha & \alpha & 0 & \cdots & 0 & 0 & 0 & 0\\ \ddots & \ddots & \ddots & \; & \cdots & 0 & 0 & 0 & 0\\ 0 & 0 & 0 & 0 & \cdots & \alpha & 2-2\alpha & \alpha & 0\\ 0 & 0 & 0 & 0 & \cdots & 0 & \alpha & 2-2\alpha & 0\\ 0 & 0 & 0 & 0 & \cdots & 0 & 0 & 0 & 0 \end{array}\right],$$

$$A={\left[4\Delta xs_{2}s_{3}s_{4}\;b^{n+1}/s_{1},0,\cdots ,0,2\alpha ,1\right]}^{T},$$

and

$${\underline{c}}^{n}={\left[c_{0}^{n},\;c_{1}^{n},c_{2}^{n},\cdots ,c_{I-2}^{n},c_{I-1}^{n},c_{I}^{n}\right]}^{T},$$

where *L* and *R* are real (I+1)×(I+1) matrices, and *A* and ${\underline{c}}^{n}$ are (I+1)-vectors.

The system describing the concentration of ferric ions through the solution can be evaluated as

$${\underline{c}}^{n+1}=L^{-1}\left(R{\underline{c}}^{n}+A\right),$$

where $c\left(0,x\right)=c_{i}^{0}=\underline{1}$. Table A1 provides the numerical values for the graphs shown in the main text. Tests were performed for various timesteps and mesh sizes to ensure numerical accuracy.

**Table A1:** Numerical values for one-dimensional simulation.

Δt	Δx	L/ℓ
0.005	0.1	10

## Appendix B. Algorithm for Dimensionless Two-Dimensional Model

The functions describing the ferric ion concentration c(t,x) and MOF occupancy $b(t,x_{1})$ are discritised, and the following definitions are used:

$$\begin{array}{cc} \; & c\left(t,x_{1},x_{2}\right)=c\left(t_{n},x_{i},x_{j}\right)=c_{i\;j}^{n},\;c\left(t,-L/\ell ,x_{2}\right)=c_{0\;j}^{n},\;c\left(t,L/\ell ,x_{2}\right)=c_{I\;j}^{n},\\ \; & c\left(t,-1,x_{2}\right)=c_{il\;j}^{n},\;c\left(t,1,x_{2}\right)=c_{ir\;j}^{n},\;c\left(t,x_{1},0\right)=c_{i\;jm}^{n},\\ \; & c\left(t,x_{1},-h/\ell \right)=c_{i\;0}^{n},\;c\left(t,x_{1},L/\ell \right)=c_{i\;J}^{n},\;b\left(t,x_{1}\right)=b\left(t_{n},x_{i}\right)=b_{i}^{n}. \end{array}$$

In the same way as the one-dimensional model was solved, the MOF occupancy is first updated for the next time step $b_{i}^{n+1}$ and later used to update the ferric ion concentration $c_{i\;j}^{n+1}$ at $|x_{1}|\leq 1$ and $x_{2}=0$. A Runge–Kutta 4 scheme is used to discritise $b(t,x_{1})$ at $|x_{1}|\leq 1$,

$$\begin{array}{cc} f(y) & =s_{2}\;c_{i\;jm}^{n}\left(1-y\right)-s_{2}\;s_{3}y,\;\;\mathrm{for}\;i=il,...,rl,\\ k_{1} & =\Delta t\;f\left(b_{i}^{n}\right),\;k_{2}=\Delta t\;f\left(b_{i}^{n}+\frac{k_{1}}{2}\right),\\ k_{3} & =\Delta t\;f\left(b_{i}^{n}+\frac{k_{2}}{2}\right),\;k_{4}=\Delta t\;f\left(b_{i}^{n}+k_{3}\right). \end{array}$$

At the next time step n+1 for i=il,...,ir, the MOF occupancy is

$$b_{i}^{n+1}=b_{i}^{n}+\frac{1}{6}\left(k_{1}+2k_{2}+2k_{3}+k_{4}\right),$$

where $b_{i}^{0}=0$.

We update the ferric ion concentration and use a forwards time, two-dimensional centred space scheme. The following difference formulas for the first and second derivatives were used:

$$\begin{array}{cc} \frac{\partial }{\partial t}c\left(t,x\right) & =\frac{c_{i\;j}^{n+1}-c_{i\;j}^{n}}{\Delta t},\\ \frac{\partial }{\partial x_{1}}c\left(t,x\right) & =\frac{c_{i+1\;j}^{n}-c_{i-1\;j}^{n}}{2\Delta x_{1}},\;\frac{\partial }{\partial x_{2}}c\left(t,x\right)=\frac{c_{i\;j+1}^{n}-c_{i\;j-1}^{n}}{2\Delta x_{2}},\\ \nabla^{2}c\left(t,x\right) & =\frac{c_{i+1\;j}^{n}-2c_{i\;j}^{n}+c_{i-1\;j}^{n}}{\Delta x_{1}^{2}}+\frac{c_{i\;j+1}^{n}-2c_{i\;j}^{n}+c_{i\;j-1}^{n}}{\Delta x_{2}^{2}}. \end{array}$$

Substituting the formulas into the ferric ion diffusion equation in the solution for $x_{2}\geq 0$ gives

$$\frac{c_{i\;j}^{n+1}-c_{i\;j}^{n}}{\Delta t}=s_{1}\left[\frac{c_{i+1\;j}^{n}-2c_{i\;j}^{n}+c_{i-1\;j}^{n}}{\Delta x_{1}^{2}}+\frac{c_{i\;j+1}^{n}-2c_{i\;j}^{n}+c_{i\;j-1}^{n}}{\Delta x_{2}^{2}}\right].$$

We collect terms with time step n+1 on the left and *n* on the right hand side of the equation so that the general scheme ∀i and j=jm+1:J−1 is

$$c_{i\;j}^{n+1}=\left(1-2s_{x}-2s_{y}\right)c_{i\;j}^{n}+s_{x}\left(c_{i+1\;j}^{n}+c_{i-1\;j}^{n}\right)+s_{y}\left(c_{i\;j+1}^{n}+c_{i\;j-1}^{n}\right).$$

Similarly, diffusion in the polymer ∀i and j=1:jm−1 is

$$c_{i\;j}^{n+1}=\left(1-2s_{x}D-2s_{y}D\right)c_{i\;j}^{n}+s_{x}D\left(c_{i+1\;j}^{n}+c_{i-1\;j}^{n}\right)+s_{y}D\left(c_{i\;j+1}^{n}+c_{i\;j-1}^{n}\right),$$

where $s_{x}=s_{1}\Delta t/\Delta x_{1}^{2}$ and $s_{y}=s_{1}\Delta t/\Delta x_{2}^{2}$. We need to account for the boundary conditions of the system and update the numerical scheme accordingly:

for ∀i,n, and j=J, far-field boundary condition (Equation (4)) at $x_{2}=L/\ell$,

$$c_{i\;J}^{n}=1,$$

for i=0 and j=jm+1:J−1, no-flux boundary condition (Equation (5)) for solution medium at $x_{1}=-L/\ell$,

$$c_{0\;j}^{n+1}=\left(1-2s_{x}-2s_{y}\right)c_{0\;j}^{n}+s_{x}\left(c_{1\;j}^{n}+c_{1\;j}^{n}\right)+s_{y}\left(c_{0\;j+1}^{n}+c_{0\;j-1}^{n}\right),$$

for i=0 and j=1:jm−1, no-flux boundary condition (Equation (5)) for polymer medium at $x_{1}=-L/\ell$,

$$c_{0\;j}^{n+1}=\left(1-2s_{x}D-2s_{y}D\right)c_{0\;j}^{n}+s_{x}D\left(c_{1\;j}^{n}+c_{1\;j}^{n}\right)+s_{y}D\left(c_{0\;j+1}^{n}+c_{0\;j-1}^{n}\right),$$

for i=I and j=jm+1:J−1, no-flux boundary condition (Equation (5)) for solution medium at $x_{1}=L/\ell$,

$$c_{I\;j}^{n+1}=\left(1-2s_{x}-2s_{y}\right)c_{I\;j}^{n}+s_{x}\left(c_{I-1\;j}^{n}+c_{I-1\;j}^{n}\right)+s_{y}\left(c_{I\;j+1}^{n}+c_{I\;j-1}^{n}\right),$$

for i=I and j=1:jm−1, no-flux boundary condition (Equation (5)) for polymer medium at $x_{1}=L/\ell$,

$$c_{I\;j}^{n+1}=\left(1-2s_{x}D-2s_{y}D\right)c_{I\;j}^{n}+s_{x}D\left(c_{I-1\;j}^{n}+c_{I-1\;j}^{n}\right)+s_{y}D\left(c_{I\;j+1}^{n}+c_{I\;j-1}^{n}\right),$$

for i=1,...,I−1 and j=0, no-flux boundary condition (Equation (5)) for polymer–optic fibre at $x_{2}=-h/\ell$,

$$c_{i\;0}^{n+1}=\left(1-2s_{x}D-2s_{y}D\right)c_{i\;0}^{n}+s_{x}D\left(c_{i+1\;0}^{n}+c_{i-1\;0}^{n}\right)+s_{y}D\left(c_{i\;1}^{n}+c_{i\;1}^{n}\right),$$

for i=0 and j=0, no-flux boundary condition (Equation (5)) at $x_{1}=-L/\ell$ and $x_{2}=-h/\ell$,

$$c_{0\;0}^{n+1}=\left(1-2s_{x}D-2s_{y}D\right)c_{0\;0}^{n}+s_{x}D\left(c_{1\;0}^{n}+c_{1\;0}^{n}\right)+s_{y}D\left(c_{0\;1}^{n}+c_{0\;1}^{n}\right),$$

for i=I and j=0, no-flux boundary condition (Equation (5)) at $x_{1}=L/\ell$ and $x_{2}=-h/\ell$,

$$c_{I\;0}^{n+1}=\left(1-2s_{x}D-2s_{y}D\right)c_{I\;0}^{n}+s_{x}D\left(c_{I-1\;0}^{n}+c_{I-1\;0}^{n}\right)+s_{y}D\left(c_{I\;1}^{n}+c_{I\;1}^{n}\right),$$

for i=1,...,I−1 and j=jm, solution–polymer boundary continuity condition (Equation (3)) at $x_{2}=0$,

$$\begin{array}{cc} \frac{\partial c}{\partial t} & =s_{1}\frac{\partial^{2}c}{\partial x_{1}^{2}}+s_{1}\frac{\partial }{\partial x_{2}}\left[D_{i}\frac{\partial c}{\partial x_{2}}\right]=s_{1}\frac{\partial^{2}c}{\partial x_{1}^{2}}+\frac{s_{1}}{\Delta x_{2}}\left[\frac{\partial c_{+}}{\partial x_{2}}-D\frac{\partial c_{-}}{\partial x_{2}}\right],\\ \frac{c_{i\;jm}^{n+1}-c_{i\;jm}^{n}}{\Delta t} & =s_{1}\left[\frac{c_{i+1jm}^{n}-2c_{i\;jm}^{n}+c_{i-1\;jm}^{n}}{\Delta x_{1}^{2}}\right]+\frac{s_{1}}{\Delta x_{2}}\left[\frac{c_{i\;jm+1}^{n}-c_{i\;jm}^{n}}{\Delta x_{2}}\right]-\frac{s_{1}D}{\Delta x_{2}}\left[\frac{c_{i\;jm}^{n}-c_{i\;jm-1}^{n}}{\Delta x_{2}}\right],\\ c_{i\;jm}^{n+1} & =\left(1-2s_{x}-s_{y}\left(1+D\right)\right)c_{i\;jm}^{n}+s_{x}\left(c_{i+1\;jm}^{n}+c_{i-1\;jm}^{n}\right)+s_{y}c_{i\;jm+1}^{n}+s_{y}Dc_{i\;jm-1}^{n}, \end{array}$$

for i=0 and j=jm, solution–polymer boundary continuity condition (Equation (3)) and no-flux boundary condition (Equation (5)) at $x_{1}=-L/\ell$ and $x_{2}=0$,

$$c_{0\;jm}^{n+1}=\left(1-2s_{x}-s_{y}\left(1+D\right)\right)c_{0\;jm}^{n}+s_{x}\left(c_{1\;jm}^{n}+c_{1\;jm}^{n}\right)+s_{y}c_{0\;jm+1}^{n}+s_{y}Dc_{0\;jm-1}^{n},$$

for i=I and j=jm, solution–polymer continuity condition (Equation (3)) and no-flux boundary condition (Equation (5)) at $x_{1}=L/\ell$ and $x_{2}=0$,

$$c_{I\;jm}^{n+1}=\left(1-2s_{x}-s_{y}\left(1+D\right)\right)c_{I\;jm}^{n}+s_{x}\left(c_{I-1\;jm}^{n}+c_{I-1\;jm}^{n}\right)+s_{y}c_{I\;jm+1}^{n}+s_{y}Dc_{I\;jm-1}^{n},$$

for i=il−1 and j=jm, solution–polymer continuity condition (Equation (3)) and no-flux boundary condition (Equation (7)) at $x_{1}=-1_{-}$ and $x_{2}=0$,

$$c_{il-1\;jm}^{n+1}=\left(1-2s_{x}-s_{y}\left(1+D\right)\right)c_{il-1\;jm}^{n}+s_{x}\left(c_{il-2\;jm}^{n}+c_{il-2\;jm}^{n}\right)+s_{y}c_{il-1\;jm+1}^{n}+s_{y}Dc_{il-1\;jm-1}^{n},$$

for i=ir+1 and j=jm, solution–polymer continuity condition solution–polymer continuity condition (Equation (3)) and no-flux boundary condition (Equation (7)) at $x_{1}=1_{+}$ and $x_{2}=0$,

$$c_{ir+1\;jm}^{n+1}=\left(1-2s_{x}-s_{y}\left(1+D\right)\right)c_{ir+1\;jm}^{n}+s_{x}\left(c_{ir+2\;jm}^{n}+c_{ir+2\;jm}^{n}\right)+s_{y}c_{ir+1\;jm+1}^{n}+s_{y}Dc_{ir+1\;jm-1}^{n},$$

At the solution–MOF–polymer boundary, we use the continuity condition (Equation (3)), to find

$$c_{i\;jm-1}^{n}=\frac{-c_{i\;jm+1}^{n}+\left(1+D\right)c_{i\;jm}^{n}}{D}.$$

For i=il,...,ir and j=jm, MOF–solution flux condition (Equation (6)) at $|x_{1}|\leq 1$ and $x_{2}=0$ gives

$$\begin{array}{cc} s_{1}\left[\frac{c_{i\;jm+1}^{n+1}-c_{i\;jm-1}^{n+1}}{2\Delta x_{2}}\right] & =s_{4}s_{2}\left(c_{i\;jm}^{n+1}\left(1-b_{i}^{n+1}\right)-s_{3}b_{i}^{n+1}\right),\\ c_{i\;jm}^{n+1} & =\frac{s_{1}\left(1+D\right)c_{i\;jm+1}^{n+1}+2\Delta x_{2}Ds_{4}s_{2}s_{3}b_{i}^{n+1}}{s_{1}\left(1+D\right)+2\Delta x_{2}Ds_{4}s_{2}\left(1-b_{i}^{n+1}\right)}. \end{array}$$

Table A2 provides the numerical values for the graphs shown in the main text. Tests were performed for various timesteps and mesh sizes to ensure numerical accuracy.

**Table A2:** Numerical values for two-dimensional simulation.

Δt	$\Delta x_{1}$	$\Delta x_{2}$	L/ℓ	h/ℓ	*D*
0.0005	0.1	0.1	10	10	0.5

Unless otherwise stated, *D* takes the value found in Table A2.

## Appendix C. Results of Varying Parameters in the Two-Dimensional Model

This section contains the figures that show the effects of variation of the remaining non-dimensional parameters for the two-dimensional model.
